# Gridded population survey sampling: a systematic scoping review of the field and strategic research agenda

**DOI:** 10.1186/s12942-020-00230-4

**Published:** 2020-09-09

**Authors:** Dana R. Thomson, Dale A. Rhoda, Andrew J. Tatem, Marcia C. Castro

**Affiliations:** 1 Department of Social Statistics and Demography, University of Southampton, Building 58, Southampton, SO17 1BJ UK; 2 WorldPop, Department of Geography and Environmental Science, University of Southampton, Building 44, Southampton, SO17 1BJ UK; 3 Biostat Global Consulting, 330 Blandford Drive, Worthington, OH 43085 USA; 4 Harvard T.H. Chan School of Public Health, 677 Huntington Avenue, Boston, MA 02115 USA

**Keywords:** Census, Survey design, Household survey, LMIC, WorldPop, LandScan

## Abstract

**Introduction:**

In low- and middle-income countries (LMICs), household survey data are a main source of information for planning, evaluation, and decision-making. Standard surveys are based on censuses, however, for many LMICs it has been more than 10 years since their last census and they face high urban growth rates. Over the last decade, survey designers have begun to use modelled gridded population estimates as sample frames. We summarize the state of the emerging field of gridded population survey sampling, focussing on LMICs.

**Methods:**

We performed a systematic scoping review in Scopus of specific gridded population datasets and "population" or "household" "survey" reports, and solicited additional published and unpublished sources from colleagues.

**Results:**

We identified 43 national and sub-national gridded population-based household surveys implemented across 29 LMICs. Gridded population surveys used automated and manual approaches to derive clusters from WorldPop and LandScan gridded population estimates. After sampling, some survey teams interviewed all households in each cluster or segment, and others sampled households from larger clusters. Tools to select gridded population survey clusters include the GridSample R package, Geo-sampling tool, and GridSample.org. In the field, gridded population surveys generally relied on geographically accurate maps based on satellite imagery or OpenStreetMap, and a tablet or GPS technology for navigation.

**Conclusions:**

For gridded population survey sampling to be adopted more widely, several strategic questions need answering regarding cell-level accuracy and uncertainty of gridded population estimates, the methods used to group/split cells into sample frame units, design effects of new sample designs, and feasibility of tools and methods to implement surveys across diverse settings.

## Background

Household surveys provide insight into the distribution of health, demographics, economics, and behaviours of populations, and are a primary resource for decision-making across low- and middle-income countries (LMICs). Household survey data are used to estimate more than a quarter of the Sustainable Development Goal (SDG) indicators, to generate small area estimates (SAEs) of indicators that support decision-making in decentralized health systems [1], and to inform the distribution of development funding to, and within, LMICs. Nevertheless, as the use of household surveys has increased over the last 40 years, data accuracy has likely decayed because survey methods have not changed while population characteristics and behaviours have—drastically.

Survey sampling methods have been mature for decades [2]. The Demographic and Health Surveys (DHS) [3], Multiple Indicator Cluster Surveys (MICS) [4], and Living Standards Measurement Surveys (LSMS) [5] have collectively supported hundreds of multi-topic surveys in over 130 countries since 1980 using essentially the same methods. They follow a stratified two-stage cluster design in which first- or second-level administrative units (e.g. provinces) serve as strata. In stage one, census enumeration areas (EAs) are selected with probability proportionate to population size (PPS), and then a field-based mapping-listing activity is conducted in each selected cluster to fully list all households. In stage two, households are sampled from the full listing by an impartial central team, and interviewers return to selected households to administer questionnaires. Rapid needs assessments and public opinion surveys follow a similar design, but tend to use a faster, less-rigorous household selection protocol during stage two; rather than performing a full household listing, interviewers perform a random walk from a central point in the cluster and directly sample households in the field [6, 7]. This approach is considered less rigorous than a full listing because interviewers may consciously or sub-consciously avoid undesirable households; the protocol can result in a “main street” bias, and information needed to adjust for household sample probabilities and non-response are, generally, not collected [8].

The last 40 years have seen dramatic increases in mobility of LMIC populations, urbanisation, and socioeconomic disparities within cities [9]. The urban poorest include climate and political refugees, seasonal migrants, and rural migrants, as well as multi-generation slum dwellers, street-sleepers, and marginalized minorities [9]. Concurrently, availability of technologies (e.g., mobile phones) and new data (e.g., high-resolution satellite imagery) has rapidly increased, though few new technologies and datasets have been incorporated into standard survey practice. This mismatch has resulted in challenges to sample frame and field protocol accuracy [10, 11]. Furthermore, the SDGs have increased emphasis on disaggregated indicators [12], raising concerns about whether current survey designs are ideal for accurate SAEs, which we highlight below. To address these emerging issues, survey practitioners have begun to use modelled gridded population datasets as an alternative to census sample frames.

Gridded population datasets are estimates of the total population in small grid cells derived with a geo-statistical model using census or small area population counts and a number of other spatial datasets [13]. The cells in gridded population estimates range in size from 30 × 30 m to 1 × 1 km, and many of these datasets are free and publicly available. In gridded population sampling, grid cells are often aggregated into clusters of a desired population size, and used in place of census EAs. To contextualise gridded population sampling, we provide further background on key reasons that teams have turned to gridded population sampling, and provide an overview of gridded population datasets.

The objectives of this paper are to provide a systematic scoping review of the datasets, tools, and methods used in existing gridded population surveys in LMICs, and outline a research agenda that would equip survey designers to decide when gridded population sampling can be viable and preferable to census-based sampling. We aim to encourage new research and practices that improve the accuracy of survey data and, ultimately, to improve accuracy of health and other household survey data to better target resources toward mobile and vulnerable populations.

### Reasons for use of gridded population sampling

The main reason that survey practitioners have turned to gridded population sampling is lack of a current, accurate census sample frame. One in four LMICs has not had a census in the last 10 years [14]. High rates of urban growth and mobility in LMICs mean that megacities in Asia, and soon Africa, grow by 1000 people per day [15]. Since 2000, the average household survey sample frame in LMICs was 7 years old, with some surveys using 15 (Pakistan) and 30 (DR Congo) year old sample frames [16]. Vulnerable populations are most likely to be excluded from surveys with an outdated sample frame because population growth is greater among lower-income households, and they are more likely to be undercounted in censuses [11].

The second reason for choosing gridded population sampling is that standard survey methods, largely developed for rural settings 40 years ago, struggle to sample mobile and vulnerable households accurately [17]. Even if the census sample frame is complete and updated, a time gap between the household mapping-listing activity and interviews in DHS, MICS, LSMS, and similar surveys means that mobile and vulnerable households are more likely to be counted as non-responders or to be under-listed during survey fieldwork. Furthermore, the mappers-listers who are responsible for generating the final household sample in the DHS, MICS, LSMS, and similar surveys frame have short interactions (e.g. 5–15 min) with residents. With limited rapport, residents may be unwilling to describe informal households in the dwelling (living space, e.g. apartment), and/or the mapping-listing team assumes one household occupies each dwelling which is simply not the case in modern LMIC cities [16, 17]. In LMICs that do not have geocoded census EA boundaries, mapping-listing activities rely on hand-sketched paper maps and subjective descriptions of EA boundaries by local leaders, leading to further potential biases.

A third reason for choosing gridded population sampling is to produce improved small area estimates. In recent years, funders and decision-makers have pushed for important health outcomes to be measured at smaller administrative scales (e.g., district) for policy planning and evaluation [1, 12]. Increased availability of satellite imagery has enabled survey outcomes to be modelled at fine-scale using geostatistical SAE techniques [18]. However, SAEs based on the stratified two-stage PPS design tend to have large uncertainty in sparsely-sampled rural areas and in heterogeneous urban settings [19–21]. Gridded population estimates can provide more up-to-date and detailed population counts than outdated census frames, permit new survey designs such as area-microcensus sampling to eliminate the time lag between mapping-listing and interviews, and facilitate spatial oversampling to improve survey-based SAEs.

### Gridded population data

A number of gridded population datasets are available across LMICs (Table 1). “Top-down” datasets disaggregate census counts to grid cells, while “bottom-up” estimates are based on micro-census population counts [22]. Currently, nine sources of “top-down” estimates are available for multiple LMICs, and two sources of “bottom-up” estimates are in production for multiple LMICs [13].

**Table 1:** Summary of gridded population datasets available for LMICs

Approach	Name	Coverage	Population†	Constrained to settlements	Producer	Resolution	Years	Statistical error	Available
Top-down	Gridded Population of the World v4 (GPWv4) [26, 27]	Global	Residential	No	Columbia University—Center for International Earth Science Information Network (CIESIN)	~ 1 × 1 km	2000, 2005, 2010, 2015, 2020	No	Yes—free
	Global Human Settlement Population (GHS-POP) [28, 29]	Global	Residential	Yes	Europe Commission—Joint Research Centre (JRC)	250 × 250 m	1975, 1990, 2000, 2015	No	Yes—free
	High Resolution Settlement Layer (HRSL) [30]	140 countries	Residential	Yes	Facebook & CIESIN	~ 30 × 30 m	Various 2015–2019	No	Yes—free
	World Population Estimate (WPE) [31]	Global	Residential	Yes	ESRI	150 × 150 m	2016	No–but confidence level ranked	Yes—paid
	LandScan-Global [36]	Global	Ambient	Yes	Oak Ridge National Laboratory	~ 1 × 1 km	Annually 2000–2017	No	Yes—paid
	Demobase [37]	3 countries	Residential	Yes	United States Census Bureau	~ 100 × 100 m	Various 2003–2013	Yes—at scale of input pop	Yes—free
	WorldPop-Land Cover [32, 33]	57 countries	Residential	No	WorldPop Project	~ 100 × 100 m	Various 2010–2015	No	Yes—free
	WorldPop-Random Forest [34, 35]	69 countries	Residential	No	WorldPop Project	~ 100 × 100 m	2010, 2015, 2020	Yes—at scale of input pop	Yes—free
	WorldPop-Global [34, 35]	Global	Residential	No	WorldPop Project	~ 100 × 100 m	Annually 2000–2020	Yes—at scale of input pop	Yes—free
Bottom-up	LandScan HD [39]	23 countries	Day-time, residential, & ambient	Yes	Oak Ridge National Laboratory	~ 100 × 100 m	varying	Yes—by cell	Yes—by request‡
	GRID3 [40]	10 countries	Residential	Yes	WorldPop Project, Flowminder Foundation, CIESIN, UN Population Fund (UNFPA)	~ 100 × 100 m	varying	Yes—by cell	Yes—free

^†^Residential = night-time population, Ambient = 24 h average population

^‡^Currently available to US Federal Government and mission partners to include anyone working on US Government funded work. Expected to be fully available in Autumn 2020

#### Top-down gridded population estimates

Nearly all gridded population datasets available at the time of this writing were derived from “top-down” models which disaggregate census or other full-coverage population counts into small grid cells. These models produce “pycnophylactic” estimates such that the cell-level counts re-aggregate to the counts of input administrative data [23]. Generally input population counts are adjusted to UN population projections before modelling [24], however, this still means that countries with the greatest need for improved sample frames have the least accurate top-down gridded population datasets. Additional factors influence the accuracy of top-down modelled population estimates, namely the aggregation scale of the input census data, modelling approach, and area of the output grid cell.

##### Scale of input data

The most important factor for top-down gridded population accuracy is the aggregation scale of the model input population data (e.g., census) [25]. This is intuitive; the more detailed and accurate the input dataset, the more precise and certain the output estimates will be in small grid squares.

##### Modelling approach

The simplest top-down models assume that the population is spread evenly across grid cells within administrative units (e.g. GPWv4 [26, 27]) or are weighted by land cover types (GHS-POP [28, 29]; HRSL [30]; ESRI WPE [31]; WorldPop-Land Cover [32, 33]). These modelling techniques are more mechanical than statistical, and thus do not result in estimates of model error. These models produce reasonably accurate cell-level estimates if a highly accurate dataset of built-up areas is used to mask unpopulated areas, and the input population data is both disaggregated and recent [25], all of which are rare in LMICs.

Complex modelling techniques using multiple Earth Observation-, government-, and crowd-sourced spatial covariates (e.g., WorldPop-Random Forest [34, 35], WorldPop-Global [34, 35], LandScan-Global [36], Demobase [37]) are employed to produce substantially more accurate gridded population estimates. WorldPop-Random Forest and WorldPop-Global are 100 × 100 m datasets of the residential (night-time) population based on a regression tree machine-learning method, and are accompanied by prediction errors at the scale of the input population data [34]. Neither WorldPop-Random Forest nor WorldPop-Global datasets mask built-up areas, thus they produce small, non-zero population predictions in deserts, savannahs, and forests (e.g., 0.0001 persons per cell). WorldPop-Global incorporates changes to urban extents over time, and is modelled from a reduced set of covariates that are available globally. Demobase is a free 100 × 100 m dataset of the residential (night-time) population in three countries based on semi-automated classification of high- and medium-resolution satellite imagery, with prediction errors at the scale of the input population data [37]. LandScan-Global is an annual 1 × 1 km dataset of the “ambient” population; a 24-h average of daytime commuter population and night-time residential population [36]. This dataset is derived with a smart interpolation approach and model error estimates are not provided [36].

A common issue across all top-down gridded population datasets is that they sometimes allocate population to airports, universities, factories, and government buildings, affecting cell-level accuracy in urban areas. This misallocation may be reduced by including covariates associated with variation in urban density (e.g. building footprints), and/or covariates that represent points of interest and infrastructure where people tend not to live.

##### Area of output grid cells

The geographic size of the output cells influences estimated population accuracy at the cell-level. Generally, estimates in smaller cells have greater uncertainty, and accuracy improves with cell size. For household survey sampling, however, cell-level accuracy must be balanced against feasibility of cell size for fieldwork; in dense urban contexts, a 100 × 100 m grid cell might contain 1000 s of people. Gridded population datasets with small cells are easy to aggregate into larger units, however, complex methods are required by users to disaggregate cells that are too populous for survey field work [38].

#### Bottom-up gridded population datasets

To generate gridded population estimates in countries without a recent or accurate census, “bottom-up” models are under development to estimate population counts based on recent micro-census samples rather than full censuses [22]. These models draw on geo-statistical relationships between population density in a micro-census unit and settlement type, as well as other spatial covariates to predict population counts in un-sampled areas of the country. These census-independent gridded population estimates are produced by the GRID3 and LandScan-HD projects for multiple LMICs, and have the benefit of being constrained to settled areas [39, 40]. Other projects have resulted in a bottom-up gridded population estimate for a single country (e.g. Sierra Leone [41], Afghanistan [42]).

#### Gridded population sample frame attributes

Gridded population datasets are not provided with urban/rural classes, administrative unit names, or estimates of sub-populations because they are designed to be aggregated into any desired spatial unit. Publicly available datasets can be used to classify a gridded population dataset within a geographic information system (GIS) (e.g., ArcGIS, QGIS) or statistical program (e.g., R, Python). Urban/rural datasets include the Global Urban Footprint (GUF) [43] dataset of 85 × 85 m grid cells classified as built-up or not built-up, and the Global Human Settlement GHS-SMOD [28] dataset of 1 × 1 km grid cells classified as high-dense urban, low-dense urban, rural, and unsettled based on the GHS-POP population density and GHS-BUILT-UP datasets. Administrative boundaries are available as shapefiles through a number of initiatives including GADM [44], UN-SALB [45], and MapLibrary [46].

## Methods

We conducted a systematic scoping review in Scopus using the terms: (“gridded” OR “landscan” OR “worldpop” OR “gpw” OR “ghs-pop” OR “hrsl” OR “wpe” OR “demobase”) AND (“population” OR “household”) AND “survey”. No limits were placed on the search (e.g. year or status of publication). Article abstracts were independently screened by co-authors DRT and DAR and retained if they referred to sampling of human populations. We additionally solicited reports, websites, and articles from colleagues. DRT performed a full-text review of all screened articles and reports, and retained those that described a method, tool, or survey based on gridded population data. Retained publications were reviewed for gridded population survey details including sample frame, sample design, sample size, target population, tools, and protocols used. This review followed PRISMA-ScR guidelines (see Additional file 1). A strategic gridded population survey research agenda was iteratively developed among co-authors with feedback from survey experts in a 2 day workshop and via email.

## Results

**Fig. 1:**
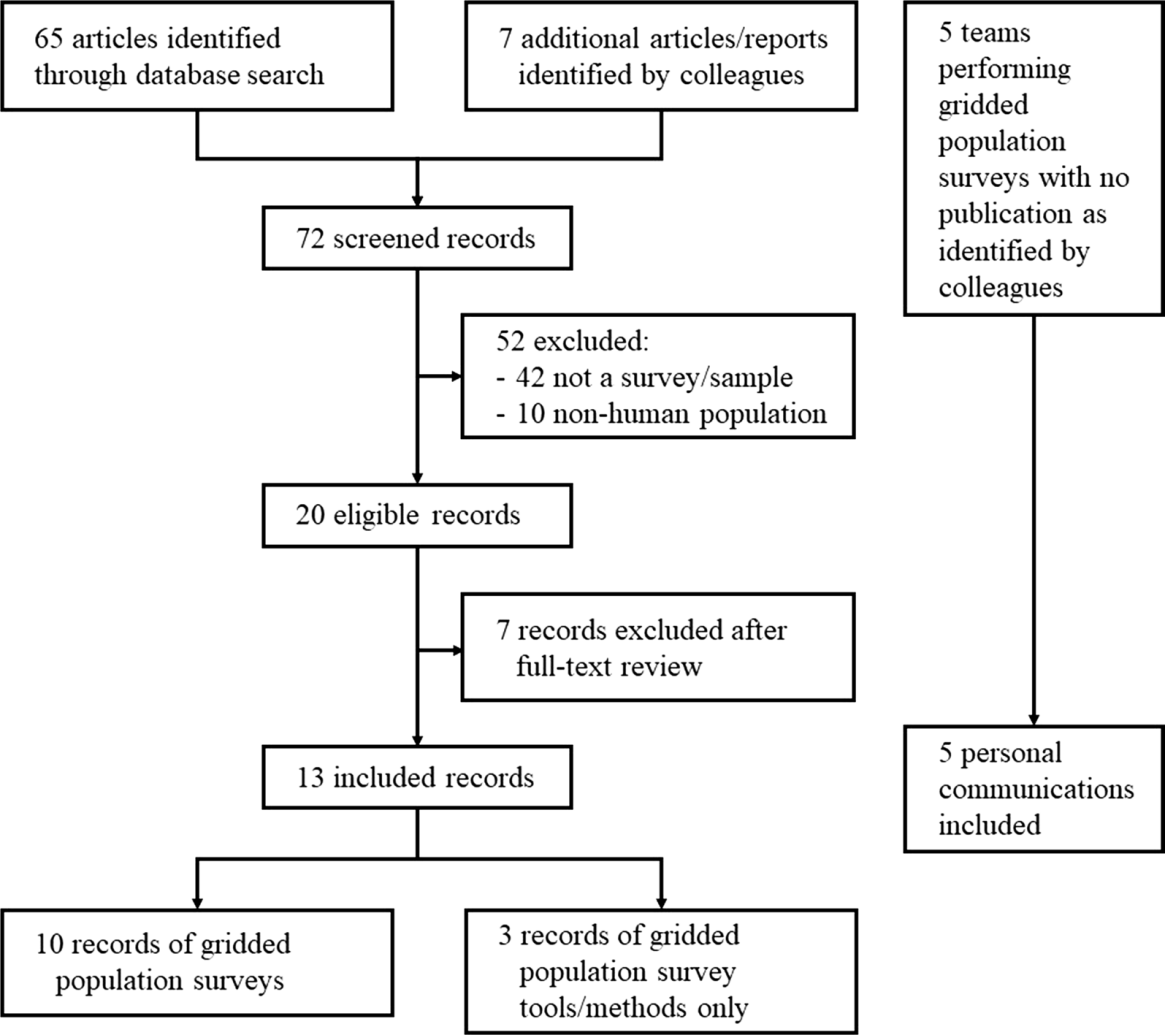
Systematic scoping review selection criteria

The review in Scopus identified 65 articles describing a gridded population survey, tool, or method. Solicitation of documents from colleagues resulted in seven additional publicly available resources, and awareness of five additional survey teams who described to us their unpublished gridded population surveys (Fig. 1). Although we did not restrict our search by geography, nearly all identified gridded population surveys were located in LMICs and were motivated by an outdated or unavailable census. This literature review resulted in 43 gridded population surveys across 29 LMICs: Bangladesh [16, 47, 48], Brazil [48], Burkina Faso, Cameroon, Colombia [48], Cote D’Ivoire, DR Congo [49, 50], Ghana [48], Guatemala [48], India [48], Indonesia, Iraq [51], Kenya [48], Mali, Mozambique [50], Myanmar [52], Nepal [16, 17, 47, 50], Niger, Nigeria [48], Rwanda [48], Somalia [53, 54], Tanzania, Thailand [48], Togo, Uganda [48], Uruguay, and Vietnam [16, 47] (Table 2). Additional gridded population surveys were conducted in Greece, Italy, and Slovenia, but excluded from this analysis (personal communication, S. Nichols, Gallup, 14 Jan 2020). Three resources described tools or methods for selecting gridded population survey clusters [38, 55, 56].

**Table 2:** Summary of gridded population surveys including their designs

Country & year	Design: coverage, strata, stages	Cluster & household sample size	Gridded population dataset	Target population, main topic(s)
DR Congo 2010 [49]	Idjwi Island, no strata, area-microcensus	50 clusters, 2078 HHs	2001 LandScan-Global	All women age 18–50, maternal and child health
Myanmar 2010 [52]	Chin state, urban/rural strata, multi-stage (spin-the-pen)	90 clusters, 720 HHs	2005 LandScan-Global (rural only)	Household head age 18 + , health and human rights
Iraq 2011 [51]	National, governorates strata, multi-stage (random-walk)	100 clusters, 1960 HHs	2008 LandScan-Global	Household head age 18 + , mortality
Bangladesh 2014–15 [48]	National, division × urbanicity strata, area-microcensus	148 clusters, 3296 HHs	2012–2016 LandScan-Global	Adult age 18 + , topics not reported
Brazil 2014–15 [48]	National, region × poverty strata, area-microcensus	149 clusters, 3652 HHs		
Colombia 2014–15 [48]	National, region × poverty strata, area-microcensus	152 clusters, 2706 HHs		
Colombia 2014–15 [48]	National, region × poverty strata, area-microcensus	152 clusters, 3037 HHs		
Ghana 2014–15 [48]	National, region × poverty × urbanicity strata, area-microcensus	151 clusters, 3113 HHs		
Guatemala 2014–15 [48]	National, department × urbanicity strata, area-microcensus	211 clusters, 3057 HHs		
India 2014–15 [48]	Three states, district × urbanicity strata, area-microcensus	467 clusters, 10,824 HHs		
Kenya 2014–15 [48]	National, province × poverty strata, area-microcensus	143 clusters, 3364 HHs		
Nigeria 2014–15 [48]	National, region × poverty strata, area-microcensus	147 clusters, 3042 HHs		
Rwanda 2014–15 [48]	National, province × poverty strata, area-microcensus	150 clusters, 3096 HHs		
Thailand 2014–15 [48]	National, region × poverty strata, area-microcensus	150 clusters, 3136 HHs		
Thailand 2014–15 [48]	National, region × poverty strata, area-microcensus	150 clusters, 3275 HHs		
Uganda 2014–15 [48]	National, region strata, area-microcensus	146 clusters, 3075 HHs		
Nepal 2015 [17]	Kathmandu Valley, no strata, multi-stage	90 clusters, 1,310 HHs (planned)	2014 WorldPop-RF	Woman age 18 + , maternal and child health
Mozambique 2017 [50]	Six districts, district strata, area-microcensus	234 clusters, 4998 HHs	2017 WorldPop-RF	Caregiver of child age 12–18, child health
DR Congo 2017 [50]	Kinshasa, communes strata, area-microcensus	210 clusters, 1,850 HHs	Bespoke derived from administrative records	Household head, food insecurity
Somalia 2017 [53, 54]	National, region × urbanicity, multi-stage	405 clusters, 6,284 HHs	Modified 2015 WorldPop-LC	Household head, economic
Nepal 2017 [16, 47, 50]	Kathmandu valley, no geographic strata, area-microcensus & multi-stage	60 clusters, 1200 HHs	2017 WorldPop-RF	Adult age 18 + , economic and non-communicable disease
Bangladesh 2018 [16, 47]	Two communities, community strata, area-microcensus	20 clusters, 400 HHs	2020 WorldPop-RF	
Vietnam 2018 [16, 47]	Long Bien District, no strata, area-microcensus	20 clusters, 400 HHs		
Colombia 2017^a^	National, region × urbanicity, two-stage (random walk)	125 clusters, 1000 HHs	2015 WorldPop-RF	Adults age 15 + , topics not reported
Tanzania 2017^a^,*	National, region × urbanicity, three-stage (random walk)	400 clusters, 4000 HHs	2015 WorldPop-RF	
Uganda 2018^a^	National, region × urbanicity, two-stage (random walk)	200 clusters, 2000 HHs	2020 WorldPop-RF	
Nigeria 2018^a^	National, region × urbanicity, two-stage (random walk)	300 clusters, 3000 HHs	2020 WorldPop-RF	
Indonesia 2018^a^	National, region × urbanicity, two-stage (random walk)	400 clusters, 4000 HHs	2015 WorldPop-RF	
Colombia 2018^a^	National, region × urbanicity, two-stage (random walk)	400 clusters, 4000 HHs	2020 WorldPop-RF	
Kenya 2018^a^	National, region × urbanicity, two-stage (random walk)	200 clusters, 2000 HHs	2015 WorldPop-RF	
Ghana 2019^a^	National, region × urbanicity, two-stage (random walk)	100 clusters, 1000 HHs	2020 WorldPop-RF	
Togo 2019^a^	National, region × urbanicity, two-stage (random walk)	100 clusters, 1000 HHs	2020 WorldPop-RF	
Cote D’Ivoire 2019^a^	National, region × urbanicity, two-stage (random walk)	100 clusters, 1000 HHs	2020 WorldPop-RF	
India 2019^b^	Uttar Pradesh state, no strata, five-stage	110 clusters, 1,026 HHs	2015 WorldPop-RF	Adult age 18 + , social and political attitudes
Uruguay 2019^c^	National, region × urbanicity, two-stage (random walk)	100 clusters, 995 HHs	2019 WorldPop-Global	Adults age 18 + , public opinion
Niger 2019–2020^d^	National, region × urbanicity, two-stage (random walk)	244 clusters, 2386 HHs	2019 WorldPop-Global (constrained to settled areas)	Adults age 18 + , daily routines and economic/political opinions
Mali 2020^d^	National, region × urbanicity, two-stage (random walk)	230 clusters, 2152 HHs		
Mauritania 2020^d^	National, region × urbanicity, two-stage (random walk)	340 clusters, 3359 HHs		
Cameroon 2019–2020^d^	National, region × urbanicity, two-stage (random walk)	279 clusters, 2866 HHs		
Burkina Faso 2020^d^	National, region × urbanicity, two-stage (random walk)	326 clusters, 2942 HHs		
Senegal 2020^d^	National, region × urbanicity, two-stage (random walk)	371 clusters, 3580 HHs		
Nigeria 2020^d^	National, region × urbanicity, two-stage (random walk)	354 clusters, 3632 HHs		
Nigeria 2020^e^	Kaduna state, urbanicity, two-stage	36 clusters, 720 HHs	2020 WorldPop-Global	Adults age 15 + , nutrition, maternal and child health

*Gridded population sample frame used in second or third stage of sampling

^a^Personal communication, S. Nichols, Gallup, 14 Jan 2020

^b^Personal communication, J. Cajka, RTI, 9 Apr 2020

^c^Personal communication, S. Staveteig Ford and M. Kirwin, US Department of State, 10 Apr 2020

^d^Personal communication, C. Carter and Y. Dudaronak, ORB International, 9 Apr 2020

^e^Personal communication, R. Bhattarai, Flowminder Foundation and M. Imohi, Nigeria National Bureau of Statistics, 10 Dec 2019

Most sample frames in early gridded population surveys were derived from LandScan-Global 1 × 1 km estimates [48, 49, 51, 52], while most recent surveys derived sample frames from WorldPop 100 × 100 m estimates (Table 2) [16, 17, 47, 50, 53]. The final selection of households followed two approaches. First, all eligible households in a cluster or segment were interviewed (called area-microcensus hereafter). Second, households were sampled within clusters or segments before interviewing (called two-stage hereafter). We note whether household sampling was conducted with a robust probability method (i.e., complete mapping-listing of households before sampling households), or a non-probability method (e.g., random-walk or spin-the-pen) [8].

Thirty-two of the 43 surveys (74%) had national coverage with 1000–4000 households each (Table 2). Nineteen surveys (47%) followed an area-microcensus design (Table 2) for one of four reasons. First, area-microcensus sampling saved time and costs by eliminating, or reducing, the mapping-listing activity [47, 50]. Second, it restricted fieldwork to one visit in insecure or hard-to-reach areas [50, 51]. Third, it provided a simple field protocol and required less training of interviewers which was assumed to ensure higher data quality [49]. Fourth, in complex, dynamic urban environments, it removed the time lag between mapping-listing and interviewing, guarding against under-listing of mobile or vulnerable households, and placed responsibility for household identification with interviewers rather than mapper-listers [17, 47].

One survey compared area-microcensus and two-stage gridded population sampling in Kathmandu, Nepal, and found that when interviewers (area-microcensus) rather than the mapper-listers (two-stage) performed the household listing, non-family and single-adult households were more likely to be identified because interviewers spent substantially more time building rapport with residents in area-microcensus clusters during the interview process [16]. This study also found lower design effects for socio-economic indicators in the area-microcensus design, suggesting better identification of heterogeneous “hidden” households, though household response rates were also lower in the area-microcensus sample [16].

**Fig. 2:**
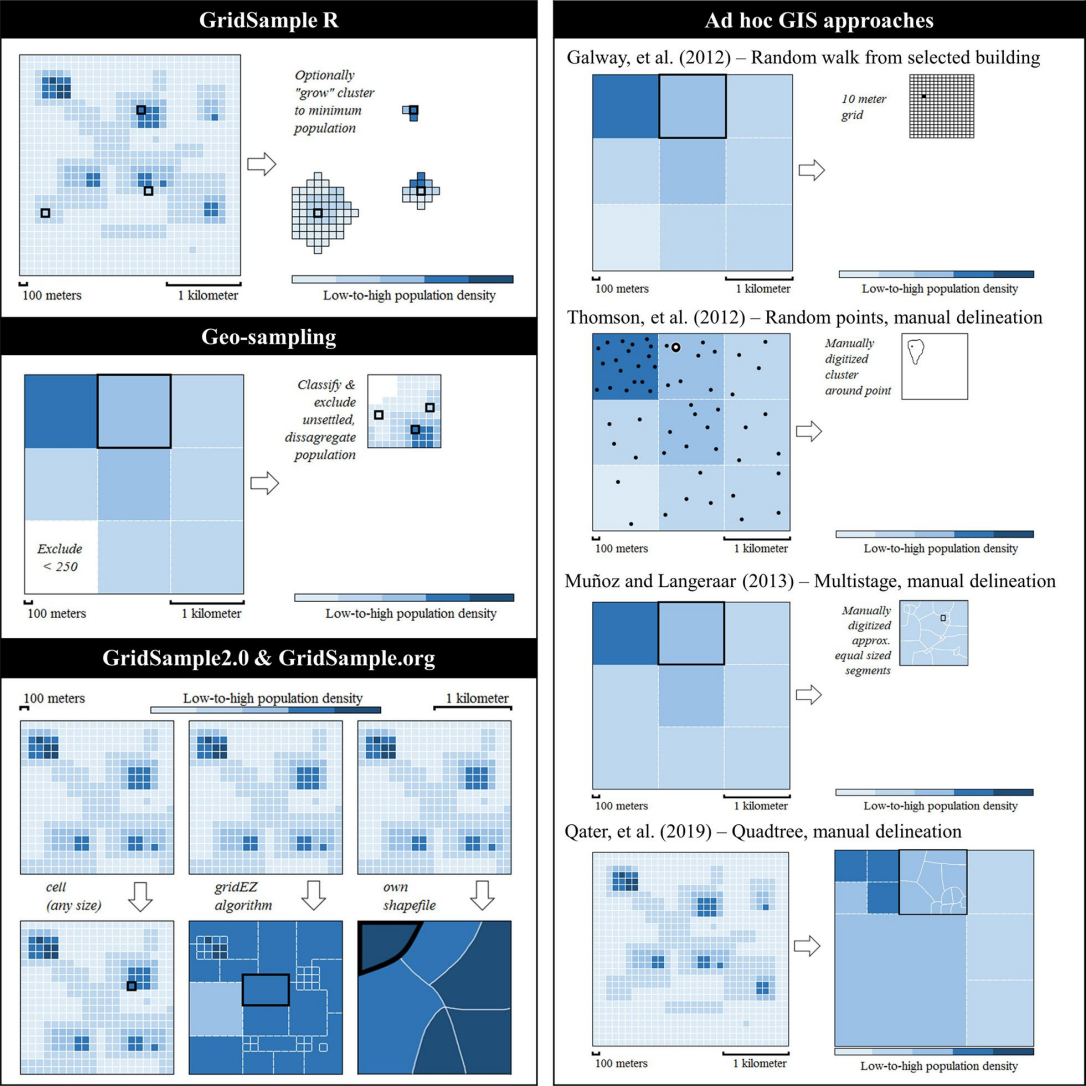
Visualisation of approaches to derive a gridded population sample frame

Four tools and numerous ad-hoc geographic information system (GIS) approaches were described to select gridded population survey clusters (Table 3), and resulted in various forms of a gridded population sample frame, visualized in Fig. 2. The first gridded population sampling tool was the open-source GridSample R package, released by Thomson and colleagues in 2016 [55] and used in six sub-national surveys [17, 47, 50]. The GridSample R algorithm treats the gridded population dataset as the sample frame and selects grid cells with PPS allowing for stratification, oversampling in urban/rural domains, and spatial oversampling [55]. The algorithm runs on a personal computer and is limited by the computer’s memory. All datasets must be pre-processed and specified by the user, allowing use of any gridded population but also requiring GIS and/or R programming skills. The algorithm enables optional “growth” of clusters to a minimum population size or maximum area by randomly adding neighbouring cells after selection of “seed” cells with PPS. While this process results in clusters with roughly consistent population size for improved fieldwork, the population counts in the “grown” clusters do not reflect the population counts used for sample selection, and may skew sample weights [55]. The output is a shapefile of cluster boundaries, with attributes of estimated population counts.

**Table 3:** Comparison of tools for gridded population sampling

Feature	GridSample R	Geo-sampling	Ad-hoc GIS	GridSample2.0	GridSample.org
Public	Yes	No	Yes	Yes	Yes
Free	Yes	No	Some	Yes	Yes
Skill level required	Advanced	Advanced	Advanced	Advanced	Basic
User selects the sample	Yes	No	Yes	Yes	Yes
Gridded pop	Any	LandScan-Global	Any	Any	WorldPop-Global
Preloaded/ provided data	No	Yes	Some	No	Yes
Pre-forms clusters	No	Yes	Some	Yes	Yes
Citations	[16, 17, 47, 50, 55]	[38, 48]	[49, 51–53, 56]	github.com/Flowminder/GridSample2.0	GridSample.org

Second, the Geo-sampling survey tool was created by RTI and used in 14 national and sub-national surveys [48] (personal communication, J. Cajka, RTI, 9 Apr 2020). The Geo-sampling tool is designed for use with large grid cells (e.g. 1 × 1 km), and supports a multi-stage stratified sampling approach. Clients are provided with a shapefile of the final cluster boundaries and population counts. In 13 surveys conducted in 2014–15, administrative units were sampled with PPS, and then 1 × 1 km LandScan-Global cells were sampled with PPS. To improve fieldwork, 1 × 1 km cells with fewer than 250 persons were excluded, potentially biasing the sample toward higher-density populations. The sampled 1 × 1 km cells were partitioned into 150, 100 or 50 m grid cells depending on population density. Next, a deep-learning residential scene classification model was used to identify and exclude small cells without settlement, and disaggregate the 1 × 1 km population to remaining small cells. Finally, three of the small cells were selected at random for an area-microcensus sample [38]. In a 2019 RTI survey, WorldPop-Random Forest estimates were aggregated to 400 × 400 m cells and used in place of 1 × 1 km cells, and a machine-learning building feature extraction algorithm was used to sample structures in the final stage of sampling (personal communication, J. Cajka, RTI, 9 Apr 2020).

Third, many gridded population surveys have developed ad-hoc approaches to sampling using GIS software, such as ArcGIS. Galway and colleagues sampled 1 × 1 km cells with PPS, then randomly selected one household in one building and performed a random walk [51]. Thomson and colleagues converted 1 × 1 km population counts to random points, selected points at random, manually delineated clusters within cells around selected points, and performed an area-microcensus sample [49]. Muñoz and Langeraar proposed an approach for 1 × 1 km cells, though it is unclear if a survey followed [56]. In this approach, 1 × 1 km cells are aggregated to 3 × 3 km grid cells and sampled with PPS. Then 1 × 1 km grid cells are combined within selected 3 × 3 km cells to achieve a minimum population and sampled with PPS. Next, they select a 1 × 1 km (or larger) area and manually delineate segments of approximately 100 households each. One segment is randomly selected, households are listed via a mapping-listing activity, and finally a sample of households is selected [56]. Sollom and colleagues joined 1 × 1 km gridded population estimates to rural village point locations and sampled points with PPS, and then used spin-the-pen to sample households in the field [52]. Qader and colleagues used gridded population estimates to update census EA counts in urban areas where EA boundaries were available, and used a quadtree method to create different sized grid cells with approximately the same population each in rural areas [53]. The combined frame was sampled with PPS before manually segmenting and randomly selecting one household per segment [53]. Finally, Gallup polling teams aggregated 100 × 100 m WorldPop-Random Forest grid cells into larger units (e.g. 200 or 500 m cells) depending on local population density, sampled aggregated grid cells with PPS, and used satellite imagery to choose a central location from which to start a random walk (personal communication, S. Nichols, Gallup, 14 Jan 2020).

Fourth, GridSample.org is a free web-based tool released in late 2019 that runs the open-source GridSample2.0 algorithm developed by Flowminder Foundation. It provides a point-and-click interface, preloaded datasets, and guidance to enter parameters and select clusters for a gridded population survey. It also leverages gridEZ, a publicly-available algorithm, to group cells into clusters before sampling. Preloaded datasets include WorldPop-Global 100 × 100 m gridded population estimates, a bespoke version of WorldPop-Global 100 × 100 m estimates constrained to settled areas, GADM administrative boundaries, and GHS-SMOD urban/rural boundaries. All surveys are implicitly stratified by level of urbanicity; stratification and spatial oversampling are supported; and custom coverage, strata, or sample frame boundaries can be uploaded by users. GridSample.org is designed for low-bandwidth settings, running sample selection remotely on a super-computer. The user is emailed a shapefile of cluster boundaries, population estimates to calculate sample weights, and a report. The US Department of State (USDS) (personal communication, S. Staveteig Ford and M. Kirwin of USDS, 10 Apr 2020), ORB International (personal communication, C. Carter and Y. Dudaronak of ORB International, 9 Apr 2020), and the Nigerian Government (personal communication, R. Bhattarai of Flowminder Foundation and M. Imohi of the Nigeria National Bureau of Statistics, 10 Dec 2019) used GridSample.org to select national or state-level household surveys. USDS in Uruguay and the Nigerian Government used GridSample.org and WorldPop-Global 100 × 100 m grid cells to create a sample frame of “medium” gridEZ units (clusters) of approximately 500 people each, while ORB International used the tool to define “large” gridEZ units (clusters) of up to 1,200 people in a maximum area of 5 × 5 km in seven Sahel countries characterized by vast unsettled areas and low-density population. The USDS and ORB International survey used a random-walk method to sample households in the field, while the Nigerian Government performed a full listing in sampled clusters before sampling and interviewing households.

A range of simple-to-advanced tools have been used to implement gridded population surveys. Lower-tech field tools include use of paper maps displaying cluster boundaries over satellite imagery in Google Earth, and paper listing forms and questionnaires [49–51]. Higher-tech field tools include tablet-based applications for navigation [16, 48], paper field maps designed in GIS [16, 17, 50, 51, 53], and tablet-based household listing and/or questionnaires [7, 16, 17, 48, 50]. Satellite imagery was essential to all gridded populations surveys to manually segment along roads, rivers, and other features [47, 49, 56], and as a field map base layer [48–51, 53]. In some surveys, satellite imagery was used to digitize building footprints and roads in OpenStreetMap which was then displayed as a field map base layer [17, 47]. Many teams included points of interest from OpenStreetMap or GPS coordinates of recognizable intersections/structures on field maps to aid navigation [17, 47, 49, 53].

## Discussion

**Fig. 3:**
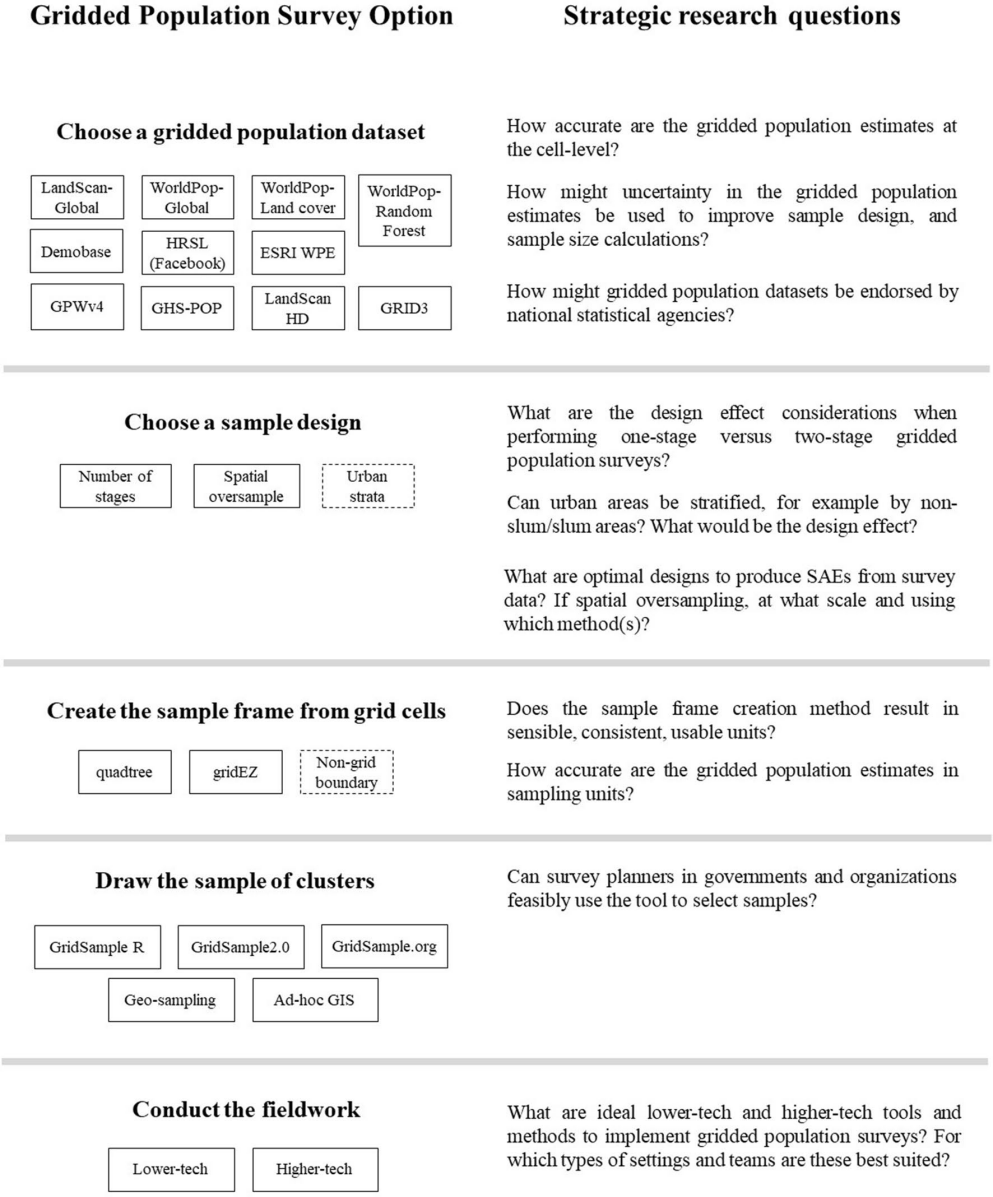
Strategic research agenda to determine when to use gridded population sampling

The successful implementation of more than 40 gridded population sample surveys across a variety of settings bodes well for this emerging field. Due to the use of English language search terms in this review, focus on academic literature, and use of gridded population sampling by some practitioners who do not publicly describe their survey methods, the number of gridded population surveys implemented is likely larger. The possibility that gridded population sampling might improve accuracy of data about vulnerable and mobile populations, especially in settings with outdated or inaccurate census data, is appealing to researchers and practitioners who work on health and social inequities in LMICs [47]. However, a survey statistician considering whether to recommend an outdated census-based frame or a gridded population frame is faced with questions about sample frame accuracy, methods to form and select sample frame units, and optimal survey designs. Next, we outline a research agenda to equip survey designers to identify situations where gridded population sampling can be a feasible and trustworthy option. The agenda shows key stages of a gridded population survey and available options (Fig. 3).

### Choose gridded population

Top-down gridded population datasets that restrict estimates to settled areas (e.g. LandScan-Global) are likely to underestimate rural, and overestimate urban, populations because small settlements are often undetected in the settlement layer. Conversely, datasets that estimate population in all landmasses (e.g. WorldPop-Global) likely overestimate rural, and underestimate urban, population because fractions of the population are allocated to unsettled cells. Factors that affect survey accuracy include the gridded population model accuracy, aggregation of the gridded population model input dataset, whether residential or ambient population is modelled, accuracy and type of covariates, and area of the cell in which population is estimated [13].

A major gap is that cell-level accuracy is not known for any top-down gridded population datasets. To assess accuracy, a recent census disaggregated to household locations would be needed, though this is rarely, if ever, available. The next best option is comparison of modelled gridded population estimates with micro-census counts from a sample of areas. Household listings from a recent geo-located household survey aggregated to cells might serve this purpose, but to our knowledge, data sharing agreements for such work have not been investigated or defined. Simulated household-level datasets are a third option [57].

Furthermore, survey designers will want to consider how uncertainty estimates might be used to improve sample designs or sample size calculations. Presently, some top-down datasets (e.g. WorldPop-Global, Demobase) include model prediction errors at the scale of the input population dataset based on internal validation, and new bottom-up datasets include cell-level uncertainty estimates. A clear understanding of cell-level accuracy is not only important to assess whether gridded population datasets are technically fit for purpose in practical applications that effects the public’s health and wellbeing [13]; this transparency is also a critical component of fostering political buy-in [58]. DHS, MICS, LSMS, and other surveys are distributed via national statistical offices, and thus their sample frames are often mandated to come from official sources. Processes are needed for national statistical agencies to engage with gridded population dataset production so that official endorsements might be made [40].

### Choose sample design

Area-microcensus sample designs in small clusters (e.g. 10–20 households) may prove to be faster and cheaper than two-stage designs in larger clusters (e.g. 100–300 households), and more accurately sample vulnerable urban populations; however, there can be a counter-balancing detriment of higher survey design effects due to variable numbers of respondents per cluster, greater within-cluster homogeneity, and lower response rates. For survey designers to assess these trade-offs and to select a sample size that will meet stakeholders’ goals for budget, timeline, and statistical precision, they need reliable projections of likely design effects in area-microcensus samples. The current limited evidence is mixed. A simulation study of a rural population in Namibia found that nearly twice as many area-microcensus clusters would be needed to achieve the same precision as a two-stage survey, holding constant the number of respondents per cluster [59]. While a study in urban Nepal found higher design effects for demographic indicators and lower design effects for socio-economic indicators in an area-microcensus design versus a two-stage design [16].

Also, as urban settlement classification becomes increasingly possible [60], survey designers need to understand how within-urban stratification affects the various sample designs used in gridded population, and other, surveys. With no way to stratify urban populations, all surveys are at risk of under-sampling or omitting slums and other vulnerable populations [61, 62]. This threat to survey accuracy and social equity will only grow as LMIC urban population continue to expand in the coming decades. In addition, research is needed to balance survey designs that can support both precise design-based estimation of outcomes and precise SAEs of indicators at fine geographic scales to support local decision-making, SDGs, and other initiatives requiring spatially disaggregated data [63].

### Create sample frame

Existing gridded population sample frame approaches result in squared-off, arbitrary cluster boundaries that are not recognizable on the ground. Improved methods are needed to use natural features such as rivers and roads to delineate cluster boundaries from gridded population data. To date, nearly all spatial feature datasets for LMICs have been produced by governments or volunteers (e.g. OpenStreetMap), neither of which are sufficiently detailed, complete, or spatially precise to support delineation of “natural” cluster boundaries across many LMICs, especially those with vast sparsely populated areas [64]. However, this is rapidly changing with new availability of very high resolution imagery and supercomputing facilities (e.g. Maxar’s building footprint and road data in 51 African countries) which might lead to new approaches to delineating “natural” cluster boundaries for gridded population data [58]. As the field continues to evolve, survey designers need to be confident that clusters will yield the right number of eligible respondents and have a geographic area that can be canvassed by a field team in the time budgeted for fieldwork.

### Draw sample

Several gridded population sampling tools and approaches are available, and their feasibility is influenced by cost, transparency of the methods, clarity of documentation, and usability by survey design professionals in government agencies and organizations who may not have advanced programming and GIS skills. The GridSample R algorithm does not scale to large geographic areas nor does include an optimal method to create clusters from grid cells, and is thus not suitable for routine national surveys. GridSample.org is free, offers ease of use and clear documentation, but currently cannot be adapted for in-house (private) use by national statistical agencies without manipulation of the underlying GridSample2.0 algorithm. Use of the Geo-sampling tool requires the hiring and support of an external company, which prohibits widespread use.

### Conduct fieldwork

The emerging field of gridded population survey sampling should recommend tools and protocols for both lower- and higher-tech settings. For example, a common protocol should be described to deal with arbitrary gridded population boundaries that intersect buildings (e.g. include buildings in north and east boundaries, exclude buildings on south and west boundaries). Uniquely, gridded population surveys rely on access to up-to-date high-resolution satellite imagery (0.5 m) for fieldwork. This is less of a challenge in urban areas worldwide thanks to Google Earth, Bing, and other free websites. However, imagery resolution in rural areas of LMICs is quite variable, with images sometimes being several years old. As a result, it would be difficult to implement gridded population surveys in areas of heavy forest or cloud cover. Furthermore, tools for implementing surveys (e.g. Survey123, OpenMapKit) tend to focus on questionnaires and often lack integration with satellite imagery, visualisation of cluster boundaries, and geo-location services in offline environments which means that multiple tools are often needed to conduct gridded survey field activities [16].

## Conclusion

Organizations with skills in GIS and digital tools can successfully implement surveys with gridded population sample frames, which have the potential to yield samples that are more representative of mobile and vulnerable respondents than outdated census-based frames. However, census-based frames are likely to be considered a safe choice by many survey designers because censuses have long been the standard and their limitations are commonly accepted. To recommend a gridded population frame would involve risks and rewards that are currently difficult to quantify. New tools are needed to evaluate gridded population datasets and frames in specific country contexts, and to facilitate low-burden survey implementation. There are opportunities to develop tools for nearly every stage of survey planning and implementation, which ultimately will improve the accuracy of survey data.

## Supplementary information

**Additional file 1.** Preferred reporting items for systematic reviews and meta-analyses extension for scoping reviews (PRISMA-ScR) checklist.

## Data Availability

Not applicable.

## Abbreviations

DHS: Demographic and Health Surveys
EA: Enumeration area
GIS: Geographic Information System
GPS: Global Positioning System
LMIC: Low- or middle-income country
LSMS: Living Standard Measurement Surveys
MICS: Multiple Indicator Cluster Surveys
PPS: Probability proportionate to (population) size
SAE: Small area estimate
SDG: Sustainable Development Goal
USDS: US Department of State
